# Laryngostroboscopic Screening in Asymptomatic Adults Undergoing Prosthetic Rehabilitation: A Prospective Observational Study

**DOI:** 10.3390/diagnostics16132004

**Published:** 2026-06-27

**Authors:** Desislava Atanasova Konstantinova, Kalina Stoyanova Georgieva-Bozhkova, Anna Kirilova Nenova-Nogalcheva, Stoyan Georgiev Katsarov

**Affiliations:** 1 Department of Material Science and Prosthetic Dental Medicine, Faculty of Dental Medicine, Medical University, 84 Tsar Osvoboditel Blvd., 9002 Varna, Bulgaria; desi.konstantinova@mu-varna.bg (D.A.K.); stoyan.katsarov@mu-varna.bg (S.G.K.); 2 Department of Oral Surgery, Faculty of Dental Medicine, Medical University, 84 Tsar Osvoboditel Blvd., 9002 Varna, Bulgaria; anna.nenova@mu-varna.bg

**Keywords:** laryngostroboscopy, vocal fold vibration, screening, asymptomatic adults, laryngeal pathology, prosthetic rehabilitation, speech function

## Abstract

**Background and Objectives:** Laryngostroboscopy is considered the gold standard for the functional assessment of vocal fold vibration and enables the detection of subtle structural and vibratory abnormalities that may not be apparent during routine examination. In interdisciplinary research involving speech analysis and prosthetic rehabilitation, exclusion of underlying laryngeal pathology is methodologically important. The aim of the present study was to evaluate the diagnostic findings obtained through laryngostroboscopic screening in asymptomatic Bulgarian adults examined within a broader research project on speech function and prosthetic rehabilitation. **Materials and Methods:** A prospective observational study was conducted between April 2022 and July 2023 at the Medical University–Varna, Bulgaria. Eighty adults without self-reported voice-related symptoms underwent laryngostroboscopic examination using an ATMOS Strobo 21 LED system (Advanced Technology Medical Systems GmbH, Lenzkirch, Germany). Participants were assessed for structural and functional laryngeal abnormalities, including alterations in movement frequency, oscillation amplitude, phase symmetry, and visible pathological changes. Descriptive statistics and chi-square tests and Fisher’s exact test analyses were used to evaluate possible associations between laryngeal pathology and demographic variables. **Results:** Normal laryngeal status was observed in 64 participants (80.0%), whereas 16 (20.0%) showed laryngostroboscopic findings. Isolated vibratory deviations were recorded separately and were not automatically classified as laryngeal pathology. Minor structural or functional variations were found in 5 participants (6.3%), functional laryngeal disorders in 6 (7.5%), benign lesions in 1 (1.3%), and diffuse inflammatory changes consistent with laryngitis in 4 (5.0%). Deviations in vibratory parameters were identified in 25 participants (31.3%) for movement frequency, 16 (20.0%) for oscillation amplitude, and 22 (27.5%) for phase synchronization. No statistically significant associations were found between laryngeal pathology and gender or age group (*p* > 0.05). **Conclusions:** Laryngostroboscopic examination identified structural and functional laryngeal findings in a proportion of asymptomatic adults recruited within a speech-function research framework. Functional vibratory deviations were observed more frequently than overt structural pathology. These findings demonstrate that previously unrecognized laryngeal abnormalities may be present even in individuals without apparent voice-related complaints. Further studies incorporating speech-function outcomes and larger cohorts are required to clarify the clinical significance of these observations.

## 1. Introduction

Laryngostroboscopy remains one of the most widely used methods for functional assessment of vocal fold vibration, allowing detailed visualization of mucosal wave propagation, amplitude, symmetry, and glottal closure patterns [1,2,3,4]. In contrast to conventional laryngoscopy, stroboscopic imaging enables the detection of subtle vibratory abnormalities that may not be clinically apparent during routine examination [5,6,7]. Owing to its high sensitivity, laryngostroboscopy plays a central role in the diagnosis and follow-up of both functional and structural voice disorders, including benign lesions, inflammatory conditions, neuromuscular dysfunction, and early malignant changes [8,9,10,11,12,13,14,15].

Voice production is a complex physiological process that depends on the coordinated interaction between the respiratory system, the larynx, and the supraglottic resonating structures of the oral cavity. Alterations in any of these components may influence phonatory performance and speech intelligibility. In prosthetic dental rehabilitation, changes in occlusion, vertical dimension, and oral cavity morphology may affect resonance and articulation patterns [16,17,18]. Consequently, when investigating speech function in patients undergoing prosthetic treatment, it is essential to exclude underlying laryngeal abnormalities that could act as confounding factors.

Epidemiological data indicate that voice disorders are relatively common in the general population, even among individuals without overt symptoms [19,20]. Moreover, studies have demonstrated that subclinical or mild laryngeal alterations may be detectable only through instrumental assessment, particularly in early inflammatory or functional disorders [21,22]. Despite the established diagnostic value of laryngostroboscopy, data regarding the prevalence and characteristics of laryngeal findings in asymptomatic adult populations remain limited, particularly in Southeastern Europe. Although videostroboscopy is widely used in patients presenting with voice complaints, relatively few studies have focused specifically on laryngostroboscopic findings in asymptomatic adults. Furthermore, available data remain heterogeneous with respect to diagnostic criteria, study populations, and definitions of clinically relevant abnormalities [23,24,25].

The present study formed part of a broader interdisciplinary research project investigating speech function and prosthetic rehabilitation. Within the overall research framework, participants subsequently underwent speech-function assessment performed by a certified speech-language pathologist using a standardized methodology adapted for Bulgarian speech. The laryngostroboscopic examination was incorporated as a preliminary screening procedure with the purpose of identifying previously unrecognized structural or functional laryngeal abnormalities that could potentially influence phonatory performance and act as confounding variables during speech-function assessment.

Although speech-function outcomes have been reported separately, the diagnostic yield of pre-assessment laryngostroboscopic screening in asymptomatic adults has not been sufficiently documented. Therefore, further characterization of structural and functional laryngeal findings in such populations may provide useful information for both clinical screening and interdisciplinary research protocols. Therefore, the aim of the present prospective observational study was to characterize the laryngostroboscopic findings detected during a pre-assessment screening protocol in asymptomatic Bulgarian adults participating in a broader speech-function research project. Specifically, the study sought to determine the frequency and nature of structural and functional laryngeal abnormalities identified by laryngostroboscopy prior to speech-function assessment.

## 2. Materials and Methods

### 2.1. Study Design and Ethical Approval

This prospective observational study was conducted between April 2022 and July 2023 at the Medical University–Varna, Bulgaria. The investigation formed part of a broader interdisciplinary research project evaluating speech function and prosthetic rehabilitation outcomes [26,27]. Within the overall research protocol, participants subsequently underwent speech-function assessment performed by a certified speech-language pathologist using a standardized methodology adapted for Bulgarian speech. The present report focuses exclusively on the laryngostroboscopic screening phase of the project.

The laryngostroboscopic examinations were performed prior to speech-function assessment in order to identify structural or functional laryngeal abnormalities that might influence phonatory performance and potentially confound the interpretation of speech-related outcomes. Subsequent speech-function assessment performed by a certified speech-language pathologist using a standardized methodology adapted for Bulgarian speech has been reported separately [28]. In addition, analyses examining the relationship between laryngeal pathology and voice function within the same research framework have also been published [29]. Therefore, the current manuscript is limited to the description of laryngostroboscopic findings identified prior to speech-function assessment. The study protocol was approved by the Ethics Committee of the Medical University–Varna (Protocol No. 116/28.04.2022) and was conducted in accordance with the Declaration of Helsinki. Written informed consent was obtained from all participants prior to examination.

### 2.2. Study Population

A total of 80 adults (41 males and 39 females) aged 18–65 years (mean age: 49.21 ± 10.13 years) were included in the study. Participants were recruited on a voluntary basis from individuals enrolled in a broader interdisciplinary research project investigating speech function and prosthetic rehabilitation.

For the purposes of the present study, asymptomatic status was defined as the absence of self-reported hoarseness, vocal fatigue, dysphonia, persistent throat discomfort, or previously diagnosed voice disorders at the time of examination. Asymptomatic status was determined solely on the basis of participant self-report. No validated voice-related questionnaires or objective clinical thresholds, including the Voice Handicap Index (VHI) or Reflux Symptom Index (RSI), were used as part of the screening protocol. None of the participants had sought otorhinolaryngological evaluation because of voice-related symptoms prior to inclusion in the study. Inclusion criteria were: age ≥ 18 years, absence of acute upper respiratory infection, absence of diagnosed laryngeal malignancy, no history of head and neck surgery within the previous six months, absence of xerostomia or uncontrolled systemic disease, absence of self-reported voice-related symptoms and ability to complete laryngostroboscopic examination.

Exclusion criteria included: current acute inflammatory conditions, previously diagnosed vocal fold paralysis, recent laryngeal surgery, inability to tolerate endoscopic examination.

Information regarding smoking status, occupational voice use, gastroesophageal or laryngopharyngeal reflux, and alcohol consumption was not systematically collected within the study protocol. Consequently, these variables were not included in the present analysis and are acknowledged as potential confounding factors.

Participants were stratified by gender and by age group (≤35 years and >35 years). The age threshold of 35 years was selected to establish two relatively balanced groups for statistical analysis. Furthermore, evidence from the literature regarding age-related changes in vocal characteristics was also taken into account [30].

### 2.3. Laryngostroboscopic Examination Protocol

All examinations were performed using an ATMOS Strobo 21 LED system (Advanced Technology Medical Systems GmbH, Lenzkirch, Germany), providing high-resolution stroboscopic visualization of vocal fold vibration. A rigid transoral laryngostroboscopic technique was used for all examinations. Video recordings were obtained during each procedure and were subsequently reviewed by the examining clinician for diagnostic documentation. All examinations and interpretations were performed by an experienced otorhinolaryngologist with extensive experience in laryngostroboscopic evaluation.

The procedure was conducted with the participant in an upright seated position. The tongue was gently stabilized, and subjects were instructed to sustain the vowel /i/ at comfortable pitch and intensity. A laryngophone synchronized the acoustic signal with the stroboscopic light source, enabling real-time visualization of apparent slow-motion vibratory cycles.

The following vibratory and morphological parameters were assessed: Fundamental vibratory frequency pattern, Oscillation amplitude, Phase symmetry, Glottal closure pattern, Presence of structural lesions, Signs of inflammatory changes.

All examinations were performed by an experienced clinician trained in laryngostroboscopic evaluation. Representative still images were extracted from recorded video sequences for documentation purposes. Because the study was designed as a descriptive screening investigation, all examinations were evaluated by a single experienced examiner. Formal intraobserver and interobserver reliability analyses were not performed.

### 2.4. Classification of Laryngeal Findings

Laryngostroboscopic findings were classified according to routinely evaluated clinical parameters commonly reported in videostroboscopic assessment, including vocal fold vibratory frequency pattern, oscillation amplitude, phase symmetry, glottal closure characteristics, structural lesions, and inflammatory changes. Interpretation was based on the overall clinical assessment of these parameters and on established descriptive criteria reported in the laryngostroboscopic literature.

Because no universally accepted quantitative scoring system exists for screening asymptomatic individuals, findings were categorized using descriptive clinical interpretation rather than a formal numerical grading scale.

Laryngeal status was classified into the following diagnostic categories:

⮚ Normal laryngeal status;
⮚ Minor structural or functional variations without evident clinical significance;
⮚ Functional laryngeal disorders;
⮚ Benign vocal fold lesions (e.g., nodules or polyps);
⮚ Diffuse inflammatory changes consistent with laryngitis.

Minor vibratory deviations in the absence of structural abnormalities were recorded separately from clinically classified pathological findings and were analyzed descriptively. The presence of an isolated vibratory deviation was not automatically classified as laryngeal pathology unless supported by additional structural or functional abnormalities during the overall clinical assessment.

For statistical purposes, findings were further dichotomized into two groups: absence of laryngeal pathology and presence of laryngeal pathology.

### 2.5. Statistical Analysis

Statistical analysis was performed using IBM SPSS Statistics (Version 17.0, IBM Corp., Armonk, NY, USA).

Descriptive statistics were calculated for demographic variables and laryngostroboscopic findings. Categorical variables were expressed as absolute values (*n*) and percentages (%). Continuous variables were presented as mean ± standard deviation (SD).

Associations between the presence of laryngeal pathology and gender or age group were evaluated using the Pearson χ^2^ test or Fisher’s exact test where appropriate. Statistical significance was defined as *p* < 0.05.

To enhance interpretability, the distribution of vibratory deviations (frequency, amplitude, and phase symmetry) was additionally analyzed descriptively.

Because the study was exploratory and descriptive in nature, no formal a priori sample size calculation was performed. The analyses were therefore intended primarily to provide preliminary prevalence estimates and to explore potential associations within the examined cohort.

## 3. Results

### 3.1. Demographic Characteristics

The study included 80 adults, comprising 41 males (51.3%) and 39 females (48.7%), aged between 18 and 65 years. The mean age of the total cohort was 49.21 ± 10.13 years. The mean age was 50.76 ± 9.41 years in males and 47.59 ± 10.72 years in females, with no statistically significant age difference between the two groups (*p* > 0.05) (Table 1).

### 3.2. Overall Laryngostroboscopic Findings

Laryngostroboscopic examination demonstrated normal laryngeal status in 64 of the 80 participants (80.0%). Pathological or clinically relevant structural/functional findings were identified in 16 participants (20.0%). Isolated vibratory deviations without sufficient accompanying findings to support a diagnosis of laryngeal pathology were recorded separately and were not included in the pathological category.

Among the detected abnormalities, minor structural or functional variations without evident clinical impact were observed in 5 participants (6.3%), functional laryngeal disorders in 6 participants (7.5%), benign vocal fold lesions in 1 participant (1.3%), and diffuse inflammatory changes consistent with laryngitis in 4 participants (5.0%).

These findings indicate that although the majority of the cohort showed no overt laryngeal pathology, one in five asymptomatic adults presented abnormalities detectable by instrumental examination.

### 3.3. Vibratory Characteristics

Analysis of vocal fold vibratory parameters revealed that normal movement frequency was present in 55 participants (68.8%), whereas 25 participants (31.3%) showed deviations from the normal vibratory frequency pattern. These vibratory findings were evaluated independently from the final diagnostic classification and therefore should not be interpreted as synonymous with clinically relevant laryngeal pathology.

Oscillation amplitude was within physiological limits in 64 participants (80.0%), while abnormal amplitude was observed in 16 participants (20.0%). Of these, 8 participants (10.0%) demonstrated increased amplitude and 8 participants (10.0%) reduced amplitude.

Phase synchronization between the vocal folds was preserved in 58 participants (72.5%), whereas 22 participants (27.5%) exhibited phase asymmetry or impaired synchronization.

Overall, vibratory deviations were observed more frequently than overt structural lesions. However, these findings should be interpreted cautiously because mild variations in vibratory behavior may occur in otherwise asymptomatic individuals and do not necessarily indicate clinically significant pathology (Table 2A,B).

### 3.4. Association Between Laryngeal Pathology and Gender

Laryngeal pathology was identified in 8 of 41 males (19.5%) and in 8 of 39 females (20.5%). No statistically significant association was found between gender and the presence of laryngeal pathology (Pearson χ^2^ test, *p* = 0.911).

Minor structural changes were slightly more frequent in males, whereas diffuse inflammatory changes were somewhat more frequent in females. However, these differences were small and did not suggest a gender-related pattern in this cohort.

### 3.5. Association Between Laryngeal Pathology and Age Group

When participants were divided by age group (≤35 years and >35 years), no statistically significant association was observed between age and the presence of laryngeal pathology (Pearson χ^2^ = 0.352, df = 1, *p* = 0.553). Fisher’s exact test likewise showed no significant association (two-sided *p* = 0.622) (Table 3).

### 3.6. Representative Laryngostroboscopic Images

Two representative still images extracted from the recorded video examinations were included to illustrate typical laryngostroboscopic findings in the examined cohort.

**Figure 1:**
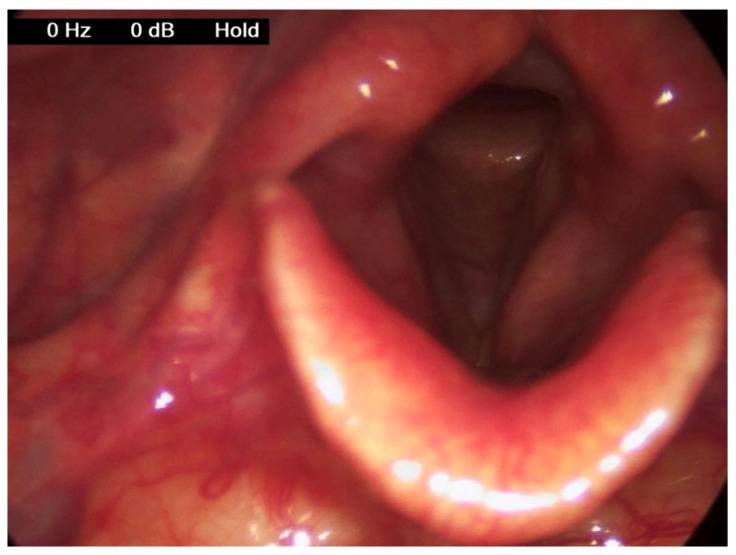
Representative laryngostroboscopic image showing normal vocal fold appearance and symmetrical vibratory pattern.

Figure 1 presents a normal laryngostroboscopic pattern, characterized by symmetrical vocal fold movement, preserved vibratory behavior, and the absence of visible structural abnormalities (Figure 1).

**Figure 2:**
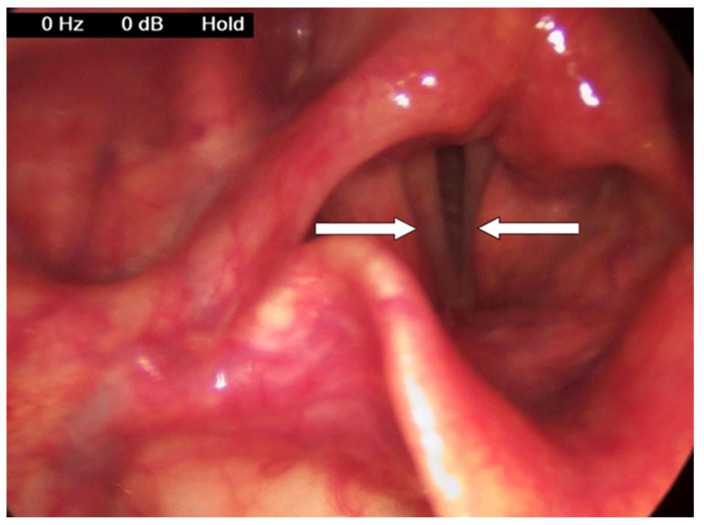
Representative laryngostroboscopic image demonstrating bilateral inflammatory mucosal changes along the medial edges of the vocal folds (white arrows), consistent with chronic laryngitis.

Figure 2 demonstrates pathological findings consistent with chronic laryngitis, including inflammatory mucosal changes and disturbed vibratory characteristics (Figure 2).

## 4. Discussion

The present study evaluated laryngostroboscopic findings in a cohort of asymptomatic Bulgarian adults examined within a broader interdisciplinary research project on speech function and prosthetic rehabilitation. The principal finding was that 20.0% of the participants demonstrated structural or functional laryngeal abnormalities despite the absence of overt clinical symptoms. In addition, deviations in vibratory parameters, particularly movement frequency and phase symmetry, were observed in a notable proportion of the cohort. The present findings indicate that structural and functional laryngeal alterations may be identified in a proportion of adults without apparent voice-related complaints. However, the clinical significance of these findings remains uncertain because the study did not include perceptual voice assessment, acoustic analysis, or patient-reported voice outcome measures. The participants included in the present screening study were recruited from a broader research program investigating speech function in prosthetic rehabilitation. Speech-function assessment performed by a certified speech-language pathologist using a methodology adapted for Bulgarian speech has been reported separately, as have analyses examining the relationship between laryngeal pathology and voice function. Therefore, the current report focuses specifically on the laryngostroboscopic screening findings obtained prior to those assessments [28,29].

Laryngostroboscopy remains one of the most informative instrumental methods for assessing vocal fold vibration, because it enables visualization of dynamic functional features that are not always detectable by routine clinical examination [2,5,6,9,10,11,12,13,30]. In the present study, most participants exhibited normal laryngeal status; however, instrumental screening identified minor structural variations, functional disturbances, benign lesions, and inflammatory changes in one-fifth of the examined adults. These findings may warrant consideration in future studies evaluating speech production, resonance, or phonatory adaptation, particularly when participants are assumed to have normal laryngeal function in the absence of instrumental examination.

The interdisciplinary context of the present investigation is particularly important. Speech production depends on the coordinated interaction between the larynx, the resonating cavities, and the articulatory structures of the oral cavity. In patients undergoing prosthetic rehabilitation, modifications in oral anatomy, occlusal relationships, and vertical dimension may influence articulation and resonance [16,17,18]. Within the broader research framework from which this cohort was derived, identification of previously unrecognized laryngeal abnormalities was considered methodologically relevant prior to speech-function assessment. Nevertheless, the present study was not designed to determine whether laryngostroboscopic screening improves speech-related outcomes or prosthetic rehabilitation results, and therefore no direct conclusions regarding its effectiveness in this context can be drawn.

Although no statistically significant association was observed between laryngeal pathology and gender or age group, these results should be interpreted cautiously. The lack of statistically significant differences may be related to the relatively limited sample size, the uneven subgroup distribution, and the small number of participants classified as having pathological findings. Consequently, the subgroup analyses should be considered exploratory in nature.

The primary objective of the present study was descriptive rather than hypothesis-testing. Accordingly, the findings provide preliminary information regarding the frequency and characteristics of laryngostroboscopic abnormalities identified in an asymptomatic adult cohort.

Vibratory deviations were observed more frequently than overt structural pathology. However, interpretation of these findings requires caution. Mild asymmetries in phase synchronization, oscillation amplitude, or vibratory frequency may occur in otherwise healthy individuals and do not necessarily represent clinically relevant disease. Because no complementary perceptual, acoustic, or aerodynamic voice assessment was performed, the functional significance of these isolated vibratory deviations cannot be determined from the present data [9,10,11,12,13].

Our observations are broadly consistent with previous reports showing that voice disorders and mild laryngeal abnormalities are not uncommon in adult populations [19,21,22]. At the same time, direct comparison with the literature should be made with caution, because many published studies include symptomatic patients, occupational voice users, or highly selected clinical populations. In contrast, the present study examined predominantly asymptomatic adults who underwent instrumental screening not because of voice complaints, but as part of a speech-related interdisciplinary protocol. This design represents one of the distinctive features of the study and provides a practical contribution to the literature on diagnostic screening.

The present findings may be of interest for future interdisciplinary studies involving speech-function assessment and prosthetic rehabilitation. However, because the current investigation did not evaluate speech outcomes directly, the extent to which the detected laryngeal findings influence speech performance remains to be established in future prospective studies.

The principal contribution of the present study is the provision of descriptive data regarding laryngostroboscopic findings in asymptomatic adults recruited within an interdisciplinary speech-function research framework. The results demonstrate that previously unrecognized structural or functional laryngeal alterations may be identified during instrumental examination even in the absence of self-reported voice-related symptoms. In addition, the observed discrepancy between clinically classified pathology and isolated vibratory deviations highlights the importance of cautious interpretation of laryngostroboscopic findings in asymptomatic populations.

Several limitations should be acknowledged. First, the study sample was relatively small and derived from a specific research setting, which limits the generalizability of the findings. Second, the participants were not recruited as a representative population-based sample, but as individuals involved in a broader project on speech function. Third, the available imaging material consisted of representative still frames extracted from recorded video examinations rather than systematically standardized image documentation for all participants. Finally, detailed analysis of potential risk factors such as smoking, occupational voice use, reflux, or environmental exposure was not included, and these variables may have influenced the observed findings. It should also be acknowledged that asymptomatic laryngopharyngeal reflux (LPR) may produce subtle inflammatory laryngeal findings detectable during laryngostroboscopic examination. Because objective reflux testing and standardized reflux-related questionnaires, such as the Reflux Symptom Index (RSI), were not included in the study protocol, the possibility that some inflammatory findings may have been influenced by unrecognized LPR cannot be excluded. Additional limitations include the absence of standardized perceptual voice evaluation, acoustic analysis, aerodynamic assessment, and validated patient-reported voice questionnaires. In addition, no quantitative image-based measurements of vocal fold vibration (e.g., amplitude-to-length ratios) were performed because they were beyond the scope of the present descriptive screening protocol. Future studies integrating objective image analysis with complementary acoustic voice assessment may provide a more comprehensive evaluation of the clinical significance of vibratory abnormalities. Furthermore, all laryngostroboscopic examinations were interpreted by a single examiner, and formal intraobserver or interobserver reliability analyses were not performed. These factors limit the interpretation of the clinical significance of the observed vibratory abnormalities. An additional limitation is that quantitative image-based measurements of vocal fold vibration (e.g., amplitude-to-length ratios) and complementary acoustic voice analyses were not performed because they were beyond the scope of the present descriptive screening protocol. Future studies integrating laryngostroboscopic imaging with objective quantitative parameters and acoustic voice assessment may provide a more comprehensive evaluation of the clinical significance of vibratory abnormalities.

Despite these limitations, the present study provides preliminary diagnostic data on laryngostroboscopic findings in asymptomatic Bulgarian adults and highlights the relevance of instrumental screening in interdisciplinary speech-related research. The results suggest that clinically unapparent laryngeal abnormalities may be identified in a meaningful proportion of adults prior to prosthetic or speech-function assessment. Future studies with larger cohorts and more detailed risk-factor profiling are warranted to confirm these observations and to better define the role of laryngostroboscopy in pre-prosthetic and speech-oriented diagnostic protocols.

## 5. Conclusions

Laryngostroboscopic examination identified structural and functional laryngeal findings in a subset of asymptomatic adults participating in a broader speech-function research project. In addition to overt pathological findings, isolated vibratory deviations were observed in a considerable proportion of participants. Because no complementary voice assessment was performed, the clinical significance of these abnormalities remains uncertain.

The present study provides descriptive data regarding laryngostroboscopic findings in asymptomatic adults and demonstrates that previously unrecognized laryngeal alterations may be detected during instrumental examination. Further studies incorporating standardized voice assessment, larger cohorts, and longitudinal follow-up are needed to clarify the functional relevance of these findings and their potential implications for speech-related research.

## Figures and Tables

**Table 1:** Distribution of patients by gender and age.

Group	Sample Size (*n*)	Mean Age	Standard Deviation (S)	Max Error (E)	95% Confidence Interval
Total	80	49.21	10.13	10.13	49.21 ± 2.25
Male	41	50.76	9.41	2.97	50.76 ± 2.97
Female	39	47.59	10.72	3.47	47.59 ± 3.47

**Table 2:** (A). Clinically classified laryngeal findings (*n* = 80). (B). Distribution of vibratory deviations recorded during laryngostroboscopic examination (*n* = 80).

(A)		
Finding	*n*	%
Normal laryngeal status	64	80.0
Minor structural/functional variations	5	6.3
Functional laryngeal disorders	6	7.5
Benign vocal fold lesions	1	1.3
Inflammatory changes (laryngitis)	4	5.0
Total pathological findings	16	20.0
(B)		
Parameter	Normal *n* (%)	Altered *n* (%)
Movement frequency	55 (68.8)	25 (31.3)
Oscillation amplitude	64 (80.0)	16 (20.0)
Phase synchronization	58 (72.5)	22 (27.5)

Note: Altered vibratory parameters were recorded descriptively and were analyzed separately from the final diagnostic classification. The presence of an isolated vibratory deviation was not considered sufficient for classification as laryngeal pathology.

**Table 3:** χ^2^ test for independence between the presence of LP (laryngeal pathologies) and the age of the patients studied.

Test	Valid Value	Degrees of Freedom	*p*-Value	*p*-Value (Bilateral)	*p*-Value (One-Sided)
Pearson Chi-Square	0.352 ^a^	1	0.553	—	—
Continuity correction	0.010	1	0.921	—	—
Maximum likelihood	0.325	1	0.569	—	—
Fisher exact test	—	—	—	0.622	0.427
Linear-by-linear association	0.348	1	0.555	—	—
Sample size	80	—	—	—	—

^a^. Two cells (50.0%) had expected counts below 5; therefore, Fisher’s exact test was also reported.

## Data Availability

The datasets generated and/or analyzed during the current study are not publicly available due to ethical and personal data protection restrictions but are available from the corresponding author on reasonable request and with permission of the Ethics Committee of the Medical University–Varna.

## Abbreviations

The following abbreviations are used in this manuscript:

ATMOS	Advanced Technology Medical Systems
IBM	International Business Machines
LED	Light Emitting Diode
LPR	laryngopharyngeal reflux
SPSS	Statistical Package for the Social Sciences
RSI	Reflux Symptom Index
VHI	Voice Handicap Index
χ^2^	Chi-square test

## References

[1] Lechien J.R., Saussez S., Harmegnies B., Finck C., Burns J.A. (2017). Laryngopharyngeal reflux and voice disorders: A multifactorial model of etiology and pathophysiology. J. Voice.

[2] Kaszuba S.M., Garrett C.G. (2007). Strobovideolaryngoscopy and laboratory voice evaluation. Otolaryngol. Clin. N. Am..

[3] Bonilha H.S., Focht K.L., Martin-Harris B. (2015). Rater methodology for stroboscopy: A systematic review. J. Voice.

[4] Kelley R.T., Colton R.H., Casper J., Paseman A., Brewer D. (2011). Evaluation of stroboscopic signs. J. Voice.

[5] Brockmann-Bauser M., de Paula Soares M.F. (2023). Do we get what we need from clinical acoustic voice measurements?. Appl. Sci..

[6] Oliveira R., Gama A., Magalhães M. (2021). Fundamental voice frequency: Acoustic, electroglottographic, and accelerometer measurement in individuals with and without vocal alteration. J. Voice.

[7] Hoffman J., Barańska M., Niebudek-Bogusz E., Pietruszewska W. (2025). Comparative Evaluation of High-Speed Videoendoscopy and Laryngovideostroboscopy for Functional Laryngeal Assessment in Clinical Practice. J. Clin. Med..

[8] Marshall C., Lyons T., Omari A.A., Alnouri G., Sataloff R.T. (2025). Misdiagnosis of vocal fold nodules. J. Voice.

[9] Zarachi A., Tafiadis D., Exarchakos G., Lianou A.N., Liontos A., Psychogios G. (2023). The Utility of Stroboscopy in Evaluating Patients with Benign Vocal Fold Lesions. Maedica.

[10] Leduchowska A., Morawska J., Pietruszewska W. (2022). Videolaryngoendoscopic and stroboscopic evaluation in predicting the malignancy risk of vocal fold leukoplakia. J. Clin. Med..

[11] Sachdeva K., Mittal N., Sachdeva N. (2020). Role of video laryngostroboscopy in benign disease of larynx. Indian J. Otolaryngol. Head Neck Surg..

[12] Chou C.-H., Chen C.-H., Chen A.W.-G. (2024). The application of i-scan imaging for evaluating benign vocal lesions. Diagnostics.

[13] Dedivitis R.A., Castro M.A.F., Guimarães A.V., Trindade C.P. (2025). Comparison between rigid telescopic and flexible fiberoptic laryngostroboscopy. Braz. J. Otorhinolaryngol..

[14] Castro M.A., Dedivitis R.A., Pfuetzenreiter Júnior E.G., Barros A.P., Queija D.S. (2012). Videolaryngostroboscopy and voice evaluation in patients with rheumatoid arthritis. Braz. J. Otorhinolaryngol..

[15] Suda A., Sikdar A., Nivsarkar S., Phatak S., Agarwal R. (2024). Reflux Symptom Index (RSI), Videolaryngostroboscopy and Voice Analysis: A Triad of Non-Invasive Tools to Study Treatment Outcomes of Laryngopharyngeal Reflux Disease (LPRD). Indian J. Otolaryngol. Head Neck Surg..

[16] Bulycheva E., Trezubov V., Alpateva U., Bulycheva D.S. (2018). Sound production in totally edentulous patients before and after prosthetic treatment. J. Prosthodont..

[17] Zou Y., Zhan D. (2015). Patients’ expectation and satisfaction with complete denture before and after the therapy. Vojnosanit. Pregl..

[18] Abduo J. (2016). Morphological symmetry of maxillary anterior teeth before and after prosthodontic planning: Comparison between conventional and digital diagnostic wax-ups. Med. Princ. Pract..

[19] Roy N., Merrill R.M., Gray S.D., Smith E.M. (2005). Voice disorders in the general population: Prevalence, risk factors, and occupational impact. Laryngoscope.

[20] Davis R.J., Exilus S., Best S., Willink A., Akst L.M. (2023). The Geographic Distribution of Videolaryngostroboscopy in the United States. J. Voice.

[21] Marchese M.R., Longobardi Y., Di Cesare T., Mari G., Terruso V., Galli J., D’aLatri L. (2022). Gender-related differences in the prevalence of voice disorders and awareness of dysphonia. Acta Otorhinolaryngol. Ital..

[22] Wang D., Li D., Mishra S.R., Lim C., Dai X., Chen S., Xu X. (2022). Association between marital relationship and multimorbidity in middle-aged adults: A longitudinal study across the US, UK, Europe, and China. Maturitas.

[23] Milstein C.F., Charbel S., Hicks D.M., Abelson T.I., Richter J.E., Vaezi M.F. (2005). Prevalence of laryngeal irritation signs associated with reflux in asymptomatic volunteers: Impact of endoscopic technique (rigid vs. flexible laryngoscope). Laryngoscope.

[24] Lundy D.S., Casiano R.R., Sullivan P.A., Roy S., Xue J.W., Evans J. (1999). Incidence of abnormal laryngeal findings in asymptomatic singing students. Otolaryngol. Head Neck Surg..

[25] Sataloff R.T., Hawkshaw M.J., Johnson J.L., Ruel B., Wilhelm A., Lurie D. (2012). Prevalence of abnormal laryngeal findings in healthy singing teachers. J. Voice.

[26] Nedelchev D.K. (2022). Videolaryngostroboscopy as a Diagnostic Method in the Clinical Practice of Otorhinolaryngologists and Physicians in Dental Medicine. Int. Bull. Otorhinolaryngol..

[27] Nedelchev D. (2024). Studies on the Changes Occurring in Speech and Masticatory Functions during Prosthetic Treatment. Ph.D. Thesis.

[28] Georgieva-Bozhkova K., Konstantinova D., Nenova-Nogalcheva A., Nedelchev D. (2025). The impact of diastema on the articulation of speech sounds. Folia Med..

[29] Nedelchev D., Milkov M., Konstantinova D., Stoykov M., Peev S., Georgieva G., Zhekov Z. (2026). Pathologic Processes in the Larynx Leading to a Change in the Voice Function. J. Craniofac. Surg..

[30] Shrikrishna B.H., Deepa G. (2025). Laryngeal morphology in voice disorders: A review of imaging and endoscopic findings. Maedica.

